# Retrospective Analysis of Pediatric Hepatoblastoma With Tumor Rupture: Experience From a Single Center

**DOI:** 10.3389/fped.2022.799307

**Published:** 2022-03-22

**Authors:** Yu-Tong Zhang, Yu-Fei Zhao, Dian-Fei Yang, Jian Chang

**Affiliations:** Department of Pediatric Oncology, The First Hospital of Jilin University, Changchun, China

**Keywords:** hepatoblastoma, tumor rupture, high-risk, interleukin-2, pediatric

## Abstract

**Purpose:**

Hepatoblastoma (HB) tumor rupture is currently considered as a high-risk factor in some risk stratification systems. This study aimed to investigate the value of HB tumor rupture in predicting the poor prognosis.

**Methods:**

The clinical data from children with high-risk HB or HB tumor rupture at our institution from October 2008 to 2017 were retrospectively reviewed and analyzed.

**Results:**

Together, 34 children with high-risk HB or HB tumor rupture were retrospected, including 25 in the high-risk group and nine in tumor rupture group. The 3-year overall survival (OS) rate in tumor rupture group was significantly higher than that of the high-risk group (100 vs. 64%, *p* = 0.0427). In tumor rupture group, seven (77.8%) of nine patients had a hemoglobin level ≤ 8 g/L and 3 of them (33.3%) had ≤ 6 g/L at the time of diagnosis. Peritoneal perfusion with interleukin-2 was implemented for each patient. At the end of the treatment, seven (77.8%) of nine patients achieved complete response (CR). No patient died at the last follow-up.

**Conclusions:**

HB tumor rupture might not be predictive of poor prognosis with the risk of peritoneal dissemination/relapse, in which peritoneal perfusion with interleukin-2 could play a role.

## Introduction

Hepatoblastoma (HB) is the most common subtype of liver tumor, which accounts for 1% of pediatric malignancies (1). With the development of contemporary state-of-the-art management of pediatric solid tumors, the risk stratification treatment strategy has been widely accepted. With regard to the outcome of pediatric HB, its 3-year overall survival (OS) has reached over 80% (2). Notably, an optimal risk stratification strategy can improve the patients' outcome and quality of life while reducing treatment-related toxicities. Therefore, it is necessary to identify the potential prognostic factor for HB to perfect the stratification system. However, this remains a challenge at present because HB is rare and the incidence of tumor rupture at diagnosis is low. Moreover, available published data are lacking (3, 4). Because the International Childhood Liver Tumors Strategy Group (SIOPEL) 4 trial has been carried out, tumor rupture at diagnosis is considered a high-risk factor by some international liver tumor study groups (2, 5, 6). Accordingly, patients with HB tumor rupture are treated by the high-risk protocol. Recently, the Children's Hepatic tumors International Collaboration (CHIC) redefined a common set of definitions on HB (7–10). Therefore, this study re-evaluated the role of tumor rupture in predicting HB prognosis.

## Patients and Methods

### Patients

Children diagnosed with high-risk HB or HB tumor rupture at our institution from October 2008 to 2017 were enrolled in this study. All clinical data were retrospectively collected by using a standard table form. In addition, the imaging data were reviewed by at least two radiologists. Risk stratification was adopted from the SIOPEL (2, 11). For our study, we classified patients with only tumor rupture as tumor rupture group. Patients with other high risk factors but without tumor rupture were classified as high-risk group. Patients with both rupture and other HR features were excluded. Thereafter, patients were divided into two groups: the high-risk group and the tumor rupture group. Biopsy was performed before chemotherapy.

### Definition of Tumor Rupture

The definition of tumor rupture was adopted from the Pediatric Hepatic International Tumor Trial (PHITT). Specifically, tumor rupture was defined as the presence of free fluid in the abdomen or pelvis at diagnosis with at least one of the following findings of hemorrhage: (1) internal complexity/septation within the fluid; (2) high-density fluid on CT (>25 Hounsfield units); (3) imaging characteristics of blood or blood degradation products on MRI; (4) heterogeneous fluid on ultrasound with echogenic debris; and (5) visible rupture/hepatic capsular defect on imaging (10).

### General Work-Up of HB Tumor Rupture

Abdominal and thoracic CT scans and/or abdominal MRI examinations were performed at diagnosis, before biopsy, and after every 2 cycles of chemotherapy. In addition, an ultrasound was done to approach to monitor bleeding. At the same time, general biochemical indexes were measured, including alpha fetoprotein (AFP), peripheral blood cell count, vital organ functions, coagulation function, and serum electrolyte levels.

### Treatment

Operative intervention was not the preferred choice to manage tumor rupture, unless there was uncontrolled active bleeding. Peritoneal perfusion with interleukin-2 (IL-2, 4.0–6.0 × 10^6^ IU/m^2^, maximum dose 10.0 × 10^6^ IU) was implemented for each patient with an interval of 2–3 days, until free fluid reached normal levels.

The vital indicators in patients were continuously monitored to maintain hemoglobin level ≥8 g/L, balanced serum electrolyte levels, and normal coagulation and organ functions. Operative intervention was necessary when the hemoglobin level was not maintained by blood transfusion after 3 days or the normal blood pressure (BP) could not be sustained or the vital organ function was lost. As for chemotherapy regimen, all children were treated by the SIOPEL3 high-risk protocol (12) or the SIOPEL4 protocol (2).

### Treatment Response

The tumor response was assessed on the basis of the imaging findings and the serum AFP level before operation according to the SIOPEL3/4 criteria. Complete response (CR) was defined as no evidence of disease with normal AFP level after adjusting for age. Partial response (PR) was defined as at least 30% decrease in the total diameter of target lesions and an associated decrease in AFP. Progressive disease (PD) was defined as at least a 20% increase in the total diameter of target lesions and/or any unequivocal increase of AFP. Stable disease (SD) was defined as tumor shrinkage less than that of PR or tumor growth less than that of PD with no significant change in AFP (5).

### Statistical Analysis

Treatment outcomes were assessed by event-free survival (EFS) and OS. The survival curves were plotted by the Kaplan–Meier method, and the confidence intervals (CIs) were calculated according to the Rothman method.

## Results

The available data of 34 children with high-risk HB or HB tumor rupture at our institution from October 2008 to 2017 were collected, including 25 in the high-risk group and nine in the tumor rupture group. Of these, three patients were excluded because they suffered from both high-risk factors and tumor rupture. The remaining 34 patients included 25 in the high-risk group and nine in the tumor rupture group. The age of patients in the high-risk group, including 11 men and 14 women, ranged from 0.2 to 15 (median, 3.8) years. In the tumor rupture group, which included three men and six women, the age range was 0.7–13 (median, 5.1) years. There was no statistical difference in age between the two groups (*p* = 0.23).

In the tumor rupture group, nine patients had PRETEXT II–IV diseases on the PRETEXT staging system (10). Seven (77.7%) patients had hemoglobin levels <8 g/L, and, of those 7 patients, three (33.3%) had <6 g/L at diagnosis. Typically, the decline of hemoglobin level of ≥2 g/L/day was suggestive of active bleeding. In addition, three (33.3%) patients suffered from hypofibrinogenemia and two (22.2%) had thrombocytopenia. The clinical characteristics of these patients are presented in Table 1. Meanwhile, two (22.2%) patients received operative intervention due to the uncontrolled peritoneal active bleeding for over 3 days, of which one patient developed peritoneal seeding at 4 months after the emergency operation. In addition, two (22.2%) patients had slightly declined blood platelet count, whereas three (33.3%) had mildly decreased serum fibrinogen concentration. There was no evidence of imbalance of serum electrolytes. No patient was diagnosed with tumor cell lysis syndrome. Before chemotherapy, a needle biopsy was performed in seven (77.8%) patients. At the end of the treatment, seven (77.8%) patients achieved CR, whereas two (22.2%) still had mildly increased AFP levels (case 3 experienced intrahepatic relapse at 6 months after the completion of chemotherapy, whereas case 2 had normal AFP level in 1 year). In total, two events (including one PD and one relapse) occurred during the median follow-up of 5.7 years, but both patients achieved second CR through a second operation and salvage chemotherapy. No patients died at the last follow-up.

**Table 1:** Clinical characteristics of patient with HB tumor rupture.

**Patient**	**Gender/age (y)**	**PRETEXT number**	**Vascular involvement**	**Histology subtype**	**AFP at diagnosis (ng/ml)**	**Tumor site**	**Extra-hepatic metastasis**	**Tumor size (mm)**	**Operative intervention for tumor rupture**	**TP or HFn**	**Nadir of Hb (g/L)**	**CR after the first-line treatment**	**Event**	**Status at last follow-up**
1	M/12	III	V1-P0	Epi/Mix	79,941	Ll/Lm	N	163 × 138 × 109	Y	HFn (+)	5.7	Y	PD	Alive, disease free (8.2 years)
2	F/0.6	III	V1-P1	Mix/Tera	2,450	Ll/Lm/Ra	N	68 × 51 × 72	Y	TP (+), HFn (+)	2.8	N	N	Alive, disease free (3.7 years)
3	F/5	IV	V2-P0	Epi/Em	>120,000	All sections	N	103 × 64 × 119	N	TP (+)	65	N	R	Alive, disease free (4.5 years)
4	F/8	III	V0-P0	Epi/ Mix	52,929	Ra/Lm	AN	92 × 52 × 94	N	–	77	Y	N	Alive, disease free (7.1 years)
5	F/3	III	V1-P1	Epi/ Fet	>120,000	Lm/Ll	N	93 × 62 × 104	N	–	73	Y	N	Alive, disease free (3.5 years)
6	F/0.9	II	V0-P0	Epi/Fet	850,00	Ra/Rp	N	30 × 40 × 37	N	–	8.5	Y	N	Alive, disease free (6.5 years)
7	M/6.7	III	V1-P0	Epi/Fet	103,000	Ra/Rp/Ll	AN	101 × 64 × 78	N	HFn (+)	5.8	Y	N	Alive, disease free (5.6 years)
8	F/4.1	III	V0-P0	Epi/Em	45,300	Ra/Lm	N	85 × 60 × 67	N	–	7.5	Y	N	Alive, disease free (5.3 years)
9	M/5.3	II	V1-P0	Epi/Mix	57,600	Ra	N	67 × 52 × 71	N	–	9.0	Y	N	Alive, disease free (7.5 years)

*M, male; F, female; V, hepatic vein; P, portal vein; Epi, epithelial; Mix, mixed; Tera, teratoid; Em, embryonal; Fet, fetal; Ll, left lateral section; Lm, left medial section; Ra, right anterior section; Rp, right posterior section; Rl, right lateral section; Y, yes; N, no; AN, abdominal node; HFn, hypofibrinogenemia; TP, thrombocytopenia; PD, progressive disease; R, relapse*.

In the high-risk group, 22 patients had PRETEXT III disease, whereas three patients had PRETEXT IV disease. The detailed characteristics of the 25 patients are presented in Supplementary Table 1. Twenty-three patients (92%) had an overall PR. One child (4%) had SD. One patient (4%) experienced tumor progression. Of the six patients with initial lung metastases, four achieved CR, one achieved PR, and one experienced SD of the lung lesions with chemotherapy alone.

Seventeen patients achieved complete resection of the liver tumor by partial hepatectomy and four patients underwent orthotopic liver transplantation, resulting in a complete tumor resection rate of 74.83% (95% CI, 68.56–99.44%).

**Figure 1:**
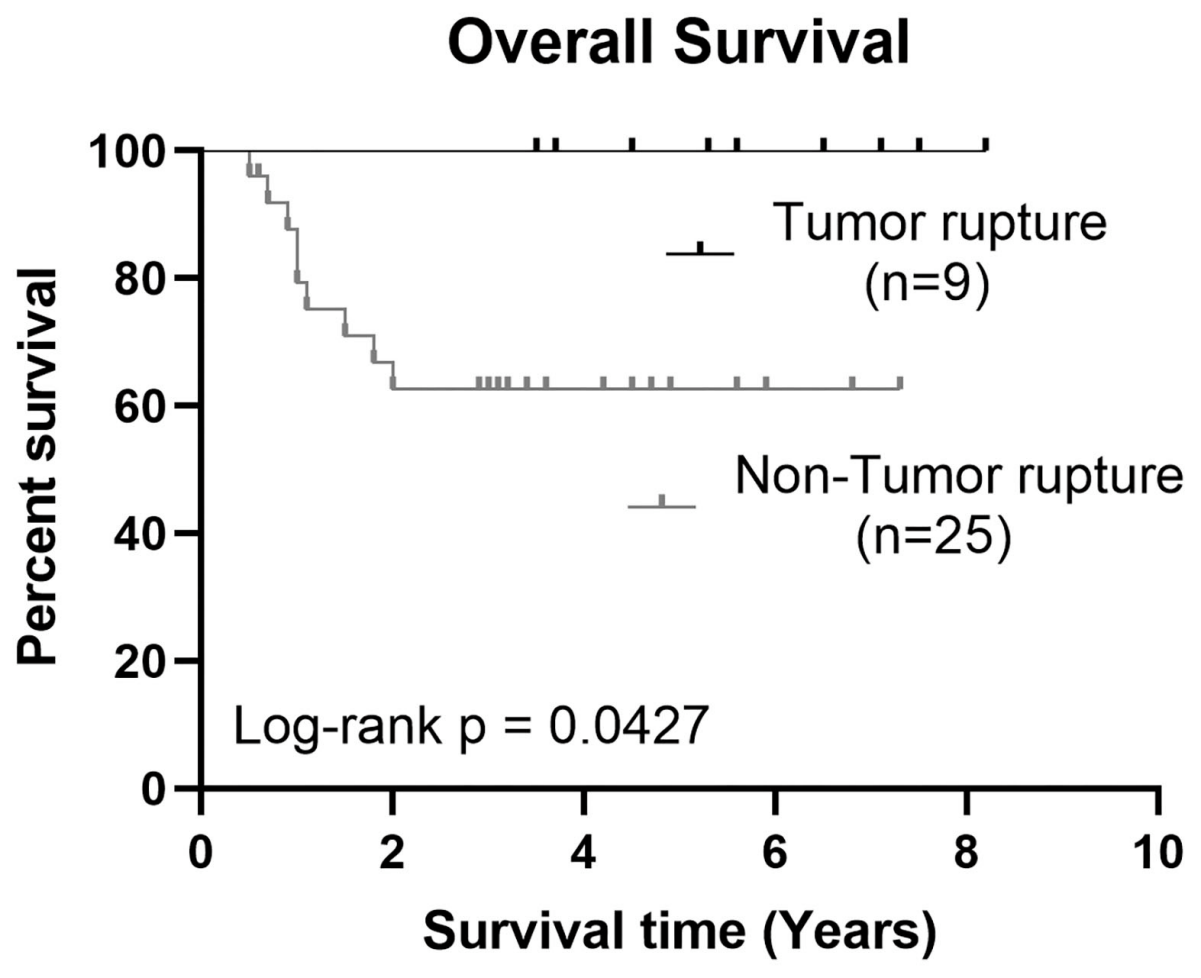
Kaplan–Meier estimates showed a significant difference of 3-year OS between HB tumor rupture group and high-risk HB group, *p* = 0.0345.

The 3-year OS and EFS in tumor rupture group were higher than those in high-risk group [100% and 68 ± 47.6.0% (95% CI, 48.35–87.65%) vs. 8,886 ± 33.3% (95% CI, 63.27–115%) and 64 ± 48.99% (95% CI, 43.78–84.22%), respectively]. The difference in OS was statistically significant (*p* = 0.0427). Additional details are shown in Figure 1.

## Discussion

HB tumor rupture at diagnosis is rare, and its incidence greatly varies among different cooperative study groups (Children's Oncology Group (COG), German Society for Pediatric Oncology and Hematology (GPOH), Japanese Study Group for Pediatric Liver Tumors (JPLT), and SIOPEL), ranging from 5 to 16% (2, 6, 13). This may be explained by the fact that no common definition on tumor rupture is available. According to the latest 2017 PRETEXT staging system, which provides a new and common set of definitions available in clinical trials, tumor rupture is clearly defined as the presence of free fluid in the abdomen or pelvis with at least one imaging criteria of hemorrhage after excluding biopsy-related hemorrhage, intraoperative rupture, non-hemorrhagic ascites, and subcapsular fluid (10). Today, no consensus has been reached on the predictive role of HB tumor rupture. In the CHIC database, tumor rupture is shown to be significantly associated with poor outcome in univariate analysis. However, it is not taken as a high-risk factor in the JPLT and the COG groups (14, 15). Considering the potential risk of peritoneal seeding after tumor rupture, patients with HB tumor rupture at diagnosis usually receive the high-risk protocol in the SIOPEL group (2).

In our retrospective study, promising results were achieved in tumor rupture group, no patient died and only two events occurred. The 3-year OS in tumor rupture group was significantly higher than that in high-risk group. These results suggest that HB tumor rupture at diagnosis might not be a poor prognostic factor in our cohort. However, there was a noticeable clue in tumor rupture group, in which the strong poor prognostic factors, such as lung metastasis, were all removed. This might partly explain the superior outcome in tumor rupture group to high-risk group. In addition, in the absence of tumor rupture, eight of nine patients in tumor rupture group had standard risk HB. In tumor rupture group, seven (77.8%) patients achieved CR at the end of the treatment, and only two showed mildly increased AFP level without residual disease on imaging. Of these two patients, one adolescent patient developed intrahepatic relapse, whereas the other infant patient gradually achieved normal AFP level. Obviously, such findings further confirmed that tumor rupture at diagnosis was not a risk factor of poor prognosis.

The management of hepatic trauma has changed significantly from the early compulsory operative approach to the non-operative management approach (16). In our cohort, we adopted the far more conservative management. After tumor rupture, in addition to the increased risk of peritoneal seeding, the injury to the biliary tree and the hepatic vasculature will result in bile and blood leakage, which present with ascites and peritonitis. The immunotherapy role in the control of malignant effusion has been validated in clinical practices (17–19), whose exact immunotherapeutic mechanism is still unknown. Oka et al. (20) reported that the immune function of cells in malignant effusions may be depressed due to a low population of cytotoxic T cells, low NK cells activity, and increased suppressor T cells, whereas the local administration of IL-2 may induce lymphokine-activated killer cells. Therefore, in our management protocol of HB tumor rupture, peritoneal perfusion with IL-2 was an obligatory procedure to protect patients from peritonitis and peritoneal seeding. In our cohort, one patient (one of nine, 11.1%) suffered from peritoneal seeding, which was partly because his postoperative chemotherapy was delayed for 4 months. After salvage chemotherapy and the second operation, the patient remained disease-free until the follow-up of 7.5 years. None of our patients experienced peritonitis or hematoma. On the basis of these results, conservative management with peritoneal perfusion of IL-2 and normal saline was an effective approach to prevent peritoneal seeding and peritonitis.

In the case of tumor rupture, blood loss to varying degrees was the most common presentation. Three (33.3%) of our patients suffered from severe anemia. In addition, the drop of hemoglobin level of ≥2 g/L/day was closely associated with potential active bleeding. The hemoglobin levels of all patients were dynamically monitored, and two patients (22.2%) received operative intervention to control bleeding. In some severe cases, the patients concomitantly presented with mild to moderate thrombocytopenia and/or hypofibrinogenemia. In our patients, the presentation of imbalanced serum electrolytes was rare, and no patient developed tumor lysis syndrome (TLS) on the basis of the laboratory criterion. TLS results from the rapid destruction of malignant cells and the abrupt release of intracellular components into the extracellular space, thus causing metabolic disorders (21). Therefore, in HB tumor rupture, the tumor cell destruction rate and the intracellular component release rate should be mild and under normal metabolic homeostasis of the body. The major challenge in HB tumor rupture should be the management of active bleeding.

The current goals of pediatric solid tumor management focuses on minimizing toxicity while maintaining excellent outcomes in low-risk disease and improving outcomes in patients with high-risk disease. In this regard, it is necessary to progressively refine the risk stratification systems and evaluate every potential annotation factor in HB. In conclusion, our management protocol of HB tumor rupture and peritoneal perfusion with IL-2 was an obligatory procedure to protect patients from peritonitis and peritoneal seeding. Consequently, HB tumor rupture might not a high-risk factor with appropriate treatment. Frankly, our results are obtained from a small sample size. Hopefully, our findings can be tested in a randomized, multi-center clinical trial in the future.

## Data Availability Statement

The original contributions presented in the study are included in the article/Supplementary Material, further inquiries can be directed to the corresponding author/s.

## Ethics Statement

The studies involving human participants were reviewed and approved by Ethical Institution of the First Hospital of Jilin University. Written informed consent to participate in this study was provided by the participants' legal guardian/next of kin.

## Author Contributions

Y-TZ, Y-FZ, and JC made substantial contributions to design of the work, drafted the manuscript, and agreed to be accountable for all aspects of the work in ensuring that questions related to the accuracy or integrity of any part of the work are appropriately investigated and resolved. D-FY made substantial contributions to the design of the work revised the manuscript critically and agreed to be accountable for all aspects of the work in ensuring that questions related to the accuracy or integrity of any part of the work are appropriately investigated and resolved. All authors have read and approved the manuscript.

## Funding

This project was supported by the Natural Science Foundation of Jilin Province, China (grant no. 182440JC010347774).

## Conflict of Interest

The authors declare that the research was conducted in the absence of any commercial or financial relationships that could be construed as a potential conflict of interest.

## Publisher's Note

All claims expressed in this article are solely those of the authors and do not necessarily represent those of their affiliated organizations, or those of the publisher, the editors and the reviewers. Any product that may be evaluated in this article, or claim that may be made by its manufacturer, is not guaranteed or endorsed by the publisher.

## Abbreviations

BP: blood pressure
CR: complete response
CIs: confidence intervals
EFS: event-free survival
HB: hepatoblastoma
OS: overall survival
PD: progression disease
PHITT: Pediatric Hepatic International Tumor Trial
PR: partial response
SD: stable disease
SIOPEL: International Childhood Liver Tumors Strategy Group
TLS: tumor lysis syndrome.
